# Drs. Hullihen and Robinson on Odontalgia

**Published:** 1850-01

**Authors:** 


### DRS. HULLIHEN AND ROBINSON ON ODONTALGIA.

In an article on alveolar abscess, published in the July number, volume second, Dental Register, we took occasion to refer to the classification of Odontalgia, as given by Dr. Robinson in his work on the teeth, published 1846. The following is the extract we then made. "Mr. Robinson, in speaking of Odontalgia, gives the following classifications: 1st, Inflammation of the Dental nerve; 2nd, fungus of the pulp; 3d, confinement of matter in the internal cavity of the tooth; 4th, disease of the Periosteum; 5th, sympathy. In inflammation of the nerve or pulp, the pain is sharp, lancenating and throbbing, and is not increased by pressure on the part.

In tooth ache, originating from the confinement of pus in the dental cavity, "pain is felt only at first when hot or cold liquids pass over the tooth; but gradually a steady gnawing pain supervenes." He then goes on to say, "that when a tooth first grows painful in this way, that it is about to suppurate; when it appears longer than the rest, loose, and exceedingly sore, the nerve has suppurated, and the pus is already oozing from the end of the fang, where the vessels and nerve enter. When the cheek begins to swell, it is a sign that the matter is spreading between the alveolus and its lining membrane, which occasions the throbbing pain felt during the formation of Alveolar Abscess."

When the article on Alveolar Abscess was penned, we had forgotten the fact that Dr. Hullihen, in 1839, in volume 1, No. 4, American Journal of Dental Science, published an article on Odontalgia, and that he made the same classification of the disease, and what is equally worthy of remark, uses throughout the entire article, much of the same language which is, years after, employed by Dr. Robinson.

The extract is then due to Dr. Hullihen, and to place the affair in its proper light, we shall give several extracts from both, and let our readers judge of the extent of similarity. Having alluded to the article as we did, we feel it due to Dr. Hullihen to thus show to whom credit belongs.

*Dr. Hullihen's Classification.*

1st, Tooth ache from exposure of the nerve.

2d. From fungus of the nerve.

3d. From confinement of pus in the internal cavity of a tooth.

4th. From a diseased state of the periosteum covering the fang.

5th. From sympathy.

Tooth ache, from the confinement of pus in the internal cavity of a tooth, occurs more frequently than any other variety, except from exposure of the nerve. At first the tooth is painful, only when hot or cold fluids pass over it, and may continue in this situation for some time. At length, a steady, gnawing pain supervenes, and then the tooth begins to get sore, and appears a little loose and longer than the rest. About this period, the pain assumes a very different character, darting from the tooth along the courses of the nerve, to the temple, ear, and side of the head, and to the

*Dr. Robinson's Classification.*

Exposure of the Dental nerve, from fungus of the pulp, from the confinement of pus in the internal cavity of a tooth.

From disease of the membrane covering the fang, (periosteum,) from sympathy, &c.

Next after tooth ache, arising from exposure of the nerve, that which depends upon confinement of pus in the dental cavity, is the most frequent. In the commencement of these cases, the pain is felt only when hot or cold liquids pass over the affected tooth; but gradually a steady gnawing pain supervenes, the tooth become sore and tender, seems a little loose and longer than the other, and pain darts from it along the nerves to the temples, the ear, the side of the head, and to the neighboring teeth in both jaws. When a tooth first grows painful in this way, it is about to suppurate; when it appears longer than the rest, loose, and exceedingly sore, teeth of both jaws on that side; within twenty-four hours the face begins to swell, and the pain changes to one of a constant throbbing description,—symptoms which indicate that an alveolar abscess is forming. When a tooth in the manner described becomes painful, the nerve is about suppurating;—when it appears longer than the rest, loose, and is very sore, the the nerve has suppurated, and the pus is beginning to ooze out at the extremity of the fang, where the vessels enter, producing much pressure, hence the sharp darting paroxysm.

When the cheek begins to swell, the matter is effusing between the alveolus and its lining membrane, and occasions the throbbing pain, which always accompanies the formation of an alveolar abscess.

The true abscess of the antrum maxillare originates in like manner, from the same cause, when the fangs are very near, or penetrate into the cavity.

There is now and then a case that differs materially from the general progress of this disease. The nerve may suppurate, and the face swell slightly, and both pain and swelling subside without the development of an abthe nerve has suppurated, and the pus is already oozing from the end of the fang, where the vessels and nerve enter.

When the cheek begins to swell, it is a sign that the matter is spreading between the alveolus and its lining membrane, which occasions the throbbing pain felt during the formation of Alveolar abscess.

It is by the same process, when the fang of a diseased tooth is near the antrum, that true abscess of that cavity takes place.

Pain, however, may exist, the nerve may suppurate, and the face swell, and both the pain and swelling again subside, without the formation of abscess; in which case the matter insinuates itself between the end of the fang and its membraneous coverings, then forming a sac about the size of a pea, or a little larger; in the course of time, this sac will burst, and discharge its pus between the fang and the alveolar process.

scess; when this occurs, the matter is insinuated between the end of the fang and membrane covering it, forming a sac about the size of a pea, and sometimes larger, which may be felt distinctly in the gum opposite the end of the fang; by pressing on it, a fullness or sensation of pressure is felt in the tooth, but in time, this sac will burst, and an alveolar abscess will be the final result."

In speaking of fungus of the nerve, Dr. Hullihen uses the following language: "It is of a deep red color, very soft, bleeds freely upon the slightest touch, is sometimes totatally insensible; at others, highly sensitive, giving much pain of an incessant character." And again he says, "it varies in size from that of a pin's head to a pea and larger."

Dr. Robinson, in speaking of fungus pulp, says: It "is a small tumor of a deep red color, either in the canal of the fang, in which case it is so minute as to be often mistaken for a nerve itself, or else in the cavity of the crown, which it generally fills, when it is present there. It is very soft, bleeds freely on the slightest touch, and varies in size from a pin's head to a large pea. Sometimes it is quite sensitive; in other cases exquisitely sensitive."

The above extracts will suffice. More might be given, but enough is *given* to show a very *close* resemblance. It would be natural, after reading both, to suppose that Dr. Robinson had just been reading Dr. Hullihen's article before he penned his own, or that he had neglected to give to Dr. H. proper credit.

The subject of Odontalgia and Alveolar Abscess, is one of far more importance than many appear to suppose. No disease in the aggregate, gives more pain, and yet but few Physicians or even Dentists treat it understandingly.—[J. T. Cin. Ed.

